# A Structural and Biochemical Model of Processive Chitin Synthesis*

**DOI:** 10.1074/jbc.M114.563353

**Published:** 2014-06-18

**Authors:** Helge C. Dorfmueller, Andrew T. Ferenbach, Vladimir S. Borodkin, Daan M. F. van Aalten

**Affiliations:** From the ‡ Division of Molecular Microbiology,; § MRC Protein Phosphorylation and Ubiquitylation Unit, College of Life Sciences, University of Dundee, Dundee DD1 5EH, United Kingdom

**Keywords:** Carbohydrate Biosynthesis, Enzyme Inhibitor, Enzyme Mechanism, Glycosyltransferase, Protein Structure

## Abstract

Chitin synthases (CHS) produce chitin, an essential component of the fungal cell wall. The molecular mechanism of processive chitin synthesis is not understood, limiting the discovery of new inhibitors of this enzyme class. We identified the bacterial glycosyltransferase NodC as an appropriate model system to study the general structure and reaction mechanism of CHS. A high throughput screening-compatible novel assay demonstrates that a known inhibitor of fungal CHS also inhibit NodC. A structural model of NodC, on the basis of the recently published BcsA cellulose synthase structure, enabled probing of the catalytic mechanism by mutagenesis, demonstrating the essential roles of the DD and Q*XX*RW catalytic motifs. The NodC membrane topology was mapped, validating the structural model. Together, these approaches give insight into the CHS structure and mechanism and provide a platform for the discovery of inhibitors for this antifungal target.

## Teaser

**Background:** Chitin synthesis is an attractive drug target in a range of organisms but is not understood at the molecular level.

**Results:** The chitooligosaccharide synthase NodC can be assayed with a novel HTS assay, and the mechanism/fold can be probed by site-directed mutagenesis and topology mapping.

**Conclusion:** NodC is a model system to probe chitin synthesis.

**Significance:** This work enables the exploitation of chitin synthesis as a drug target.

## Introduction

Fungal infections are a threat to human health worldwide. Fungal cells are protected by a unique cell wall, which is a dynamic structure consisting of proteins and polysaccharides. One essential component is chitin, a β-1,4-linked polymer of GlcNAc. Because chitin is absent in vertebrates, its synthesis is a promising target for developing specific drugs against fungal infections. Chitin synthases (CHS)^3^ utilize the nucleotide sugar donor UDP-GlcNAc and transfer the α-linked GlcNAc sugar in an inverting mechanism onto the non-reducing end of the growing acceptor oligosaccharide (1). CHS enzymes are classified in the CAZy database as belonging to the GT-2 family (2). This family contains inverting GTs such as CHS, cellulose synthases, and hyaluronan synthases. Furthermore, CHS have been categorized into classes I, II, and III according to sequence conservation (3). In *Saccharomyces cerevisiae*, three chitin synthases have been identified and characterized functionally by means of gene disruption. These studies revealed that although single disruption of any of the three *S. cerevisiae chs* genes does not affect viability, the combined deletion of *chs2* and *chs3* is lethal (4).

CHS are large, membrane integrated enzymes with multiple domains important for subcellular localization and activation. CHS contain multiple transmembrane (TM) domains that are thought to form a transport channel for the deposition of chitin on the outer membrane, similar to cellulose synthases (5). In yeast, the best characterized CHS enzyme is chitin synthase 2 (*Sc*CHS2). This enzyme consists of three domains: an N-terminal domain, a catalytic domain of the GT-2 family containing a GT-A fold, and a C-terminal transmembrane domain. *Sc*CHS2 activity appears to be regulated by proteases or posttranslational modifications (6, 7). The N-terminal domain has been shown to be highly phosphorylated *in vivo* (7). Partial proteolysis with trypsin activates CHS *in vitro*, releasing shorter CHS fragments lacking the N-terminal domain. Truncation of this domain (d193-*Sc*CHS2) does not affect enzymatic activity (6).

*Sc*CHS2 possesses several conserved sequence motifs that are essential for chitooligosaccharide synthesis. Nagahashi *et al.* (8) have identified a conserved region upstream of the first predicted TM domain. CON1 (*Sc*CHS2 residue range 490–607) contains the sequence motifs D, (E/D)D*X*, and Q(R/Q)*X*RW, which are essential for catalytic activity. Interestingly, CON1 is conserved not only among class II CHS but also found in chitooligosaccharide synthases such as the bacterial NodC proteins found in *Rhizobium* sp. and the DG42 protein from *Xenopus* (9, 10). A second conserved CHS class II region (CON2, *Sc*CHS2 residue range 748–815) is indispensable for the synthesis of long chitooligosaccharides because mutations of single residues in this region affect the ability of *Sc*CHS2 to synthesize GlcNAc oligomers longer than chitobiose (10). CON2 is predicted to be in the cytosol after the first two TM domains (10).

Several recent reviews have covered advances in the targeting of CHS for antifungal drug development (11, 12). In 1991, Cabib (13) tested the natural product inhibitors Polyoxin D, Nikkomycin Z, and Nikkomycin X and showed these to be competitive *Sc*CHS2 inhibitors. These compounds possess a chemical scaffold similar to the substrate UDP-GlcNAc and are, therefore, believed to compete for the UDP-GlcNAc binding site in the active site of CHS (12). In 2000, a series of new *Ca*CHS1 inhibitors were identified by high throughput screening and optimized by systematic chemical modifications (14). This strategy resulted in the most potent non-competitive chitin synthase inhibitor known to date, RO-09-3024, showing an IC_50_ of 0.14 nm *in vitro* and an EC_50_ of 0.07 mg/ml *versus* the human pathogen *Candida albicans* (CY1002) (15). Since then, in essence, the pursuit of CHS inhibitors has proceeded only by exploring existing chemical space because the structures of the binding modes of existing compounds remain unknown. Structural insights into the CHS active site, combined with inhibitor screening, would give rise to new opportunities to advance these existing scaffolds in antifungal drug development.

**FIGURE 1.**
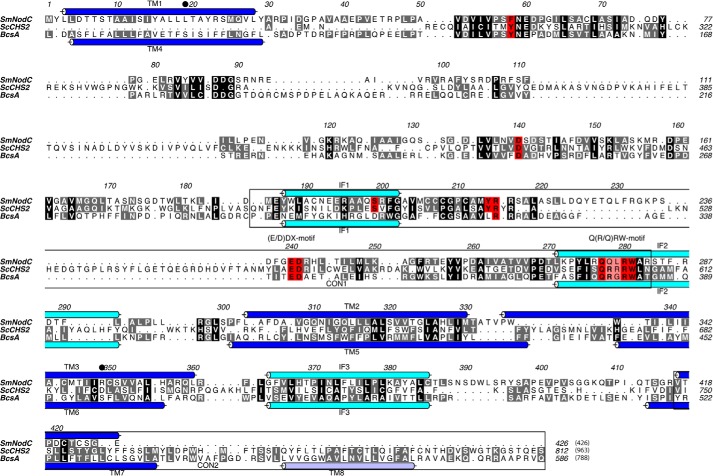
**Sequence alignment of *Sm*NodC and the catalytic domains of *Sc*CHS2 (residues 283–812) and BcsA (residues 82–574).** The whole protein sequence numbers are shown in *brackets*. TM domains and cytoplasmic IF are shown in *blue* and *light blue tubes*, respectively. The TM domains and IF for BcsA are adopted from Morgan *et al.* (5), and the TM/IF for *Sm*NodC are shown on the basis of the structural model by RaptorX (28). Conserved residues are highlighted in *black* and similar residues in *gray*. Residues that were mutated in *Sm*NodC are colored in *red*. CON1 and CON2 regions are marked with a *black box* (residues *Sc*CHS2 490–607 and 749–812). Conserved NodC residues (Leu19 and Arg-349) that block the product-binding site are labeled with *black dots*. The sequence alignment was generated using Clustal W (34) and displayed using ALINE (35).

CHS are multitransmembrane proteins that, to date, have resisted protein expression, solubilization, and crystallization for structural studies or high throughput ligand screening. A possible solution to this is to identify bacterial homologues of CHS that contain fewer transmembrane domains, are smaller, do not require eukaryotic posttranslational modifications, and can, therefore, be expressed in bacterial systems. One such apparent orthologue is the rhizobial enzyme NodC, a processive glycosyltransferase that synthesizes the chitooligosaccharide backbone of the rhizobial nodulation factor (Nod factor) essential for root nodulation of legumes (16). NodC is a β-1,4-*N*-acetylglucosamine transferase that utilizes UDP-GlcNAc as a nucleotide sugar donor and GlcNAc as the acceptor sugar to processively synthesize the Nod factor backbone, a chitooligosaccharide. NodC enzymes from different rhizobial species synthesize chitooligosaccharide backbones of specific lengths, varying from tri- to pentasaccharides (17 and reviewed in Refs. 18, 19). NodC enzymes possess striking sequence conservation with the catalytic core of CHS enzymes (Fig. 1) (20, 21). NodC proteins are smaller than CHS enzymes (∼420 amino acids in length compared with 900 to several thousand amino acids in length) because NodC enzymes lack two domains observed in chitin synthases: the N-terminal domain and the C-terminal transmembrane domain that is predicted to form a chitin transport channel across the membrane. However, the catalytic core is conserved (CON1), which contains the conserved CHS motifs (D, (E/D)D*X*, and Q(Q/R)*X*RW; Fig. 1). Topology predictions have suggested that NodC enzymes probably contain four transmembrane domains in a structural arrangement similar to that predicted for chitin synthases (22).

Here we show that bacterial NodC is a suitable model to study chitin synthases on a structural and mechanistic level. We demonstrate that NodC is inhibited by a chitin synthase inhibitor. Aided by a structural model exploiting the recently published BcsA structure, we mapped the membrane topology of *Sm*NodC and identified conserved catalytic residues for chitooligosaccharide synthesis in *Sm*NodC. The structural model provides insights into the molecular mechanism of chitin synthesis.

## EXPERIMENTAL PROCEDURES

### 

#### 

##### Cloning and Expression of SmNodC and Mutants

*Sinorhizobium meliloti* NodC (*Sm*NodC) was PCR-amplified and cloned into the pEXmCherry plasmid using the BamHI and XhoI sites. Site-directed mutagenesis was performed using the QuikChange method (Stratagene) using standard protocols. All DNA constructs were verified by DNA sequencing (The Sequencing Service, College of Life Sciences, University of Dundee, Scotland, UK). *Sm*NodC-pEXmCherry constructs were transformed into *Escherichia coli* BL21 (DE3) C43 cells. Cells were grown overnight at 37 °C in Luria-Bertani medium containing 50 μg/ml ampicillin. 10 ml of the overnight culture was used for inoculation of 1 liter of Luria-Bertani medium plus ampicillin. The bacteria were grown to an *A*_600_ of 0.6, induced by addition of 0.5 mm isopropyl-β-d-thiogalactopyranoside, and cultured for 2 h at 28 °C. Cells were harvested by centrifugation for 25 min at 3300 × *g* (4 °C). The pellet from 1 liter of culture was washed with 25 ml of chilled H_2_O and centrifuged for 25 min at 3300 × *g* (4 °C). The cells were flash-frozen in liquid nitrogen, thawed at room temperature, and resuspended in 25 ml of ice-cold buffer A (25 mm Tris-HCl (pH 7.5), 250 mm NaCl, and 2.5 mm EDTA) supplemented with 1 mg/ml of DNase and half a protease inhibitor tablet (Roche), including 2 mm DTT. The cell pellets were sonicated on ice six times for 30 s each time, with a 1-cm diameter sonicator probe (Sonya Soniprep 150). The fractions were centrifuged twice for 10 min at 12,000 × *g*, followed by a 60-min spin at 100,000 × *g*. The membrane fraction was homogenized with a Dounce homogenizer to a concentration of 25 mg/ml in 25 mm Tris-HCl (pH 7.5), 250 mm NaCl and snap-frozen in liquid nitrogen.

##### Cloning, Expression, and Investigation of SmNodC-GFP and PhoA Fusion Constructs

Full-length NodC and 11 C-terminal truncation constructs were cloned into GFP (pWaldo-d) and alkaline phosphatase (PhoA) expression vectors. *Sm*NodC-PhoA fusion proteins were expressed in *E. coli* CC118 cells. For the PhoA activity assay, 5 ml of Luria-Bertani medium + ampicillin was inoculated with 100 μl overnight culture. The cells were grown to an *A*_600_ of 0.13–0.16 at 37 °C. 1 ml of this culture was induced with 8 μl of 20% arabinose and grown to an *A*_600_ of 0.3–0.5. The cultures were treated with 4 μl of 200 mm iodoacetamide (in 10 mm Tris-HCl (pH 8.0)), incubated for 5 min at room temperature, and spun down for 20 min at 1700 g (4 °C). The cell pellet was washed with 1 ml of buffer (10 mm Tris-HCl (pH 8.0), 10 mm MgSO_4_, and 1 mm fresh iodoacetamide) and again centrifuged as described previously. The cell pellet was resuspended in 800 μl of buffer (1 m Tris-HCl (pH 8.0) and 1 mm fresh iodoacetamide). From this, 100 μl were mixed with 900 μl of activity buffer (1 m Tris-HCl (pH 8.0), 0.1 mm ZnCl_2_, and 1 mm fresh iodoacetamide). 4 μl of 0.1% SDS and 4 μl of chloroform were added and incubated for 5 min at 37 °C on a shaker (120 rpm). The samples were kept on ice, and 100 μl of 0.4% *p-*nitrophenyl phosphate were added to each reaction. This reaction was incubated for 90 min at 37 °C, and 100 μl of this solution was pipetted into a clear 96-well plate. Fluorescence was measured at excitation and emission wavelengths of 405 and 550 nm, respectively. All measurements were performed in triplicate.

The *Sm*NodC-GFP-fusion constructs were expressed in *E. coli* BL21 (DE3) C43 cells. Expression was induced at an *A*_600_ of 0.6 at 37 °C, and cells were grown for another 4 h at room temperature. A 1-ml sample of these cultures was centrifuged, and the cell pellet was resuspended in PBS. GFP fluorescence was investigated by in-gel fluorescence (23). All measurements were performed in duplicate.

##### Enzymology

The steady-state kinetics of WT *Sm*NodC-mCherry fusion were determined using a coupled enzyme assay. UDP-GlcNAc and the fluorogenic substrate 4-methylumbelliferyl-GlcNAc (4MU-GlcNAc) were obtained from Sigma. All measurements were performed in triplicate. Standard reaction mixtures consisted of 25 μg of mixed membrane fractions, 25 mm Tris-HCl (pH 7.5), 250 mm NaCl, and 5% (v/v) glycerol in a total volume of 50 μl, incubated at room temperature (20 °C). The assays were initiated by adding the mixed membrane fractions and stopped after 60 min with 50 μl of a solution containing 25 mm Tris-HCl (pH 7.5), 250 mm NaCl, and 20 mm EDTA. 0.1 μm of *Aspergillus fumigatus* chitinase B (33) was added to the reaction mixture and incubated for 60 min. The fluorescence of the released 4MU was quantified using an FLX 800 microplate fluorescence reader (Bio-Tek), with excitation and emission wavelengths of 360 and 460 nm, respectively. Metal dependence assays of WT *Sm*NodC were performed as above, with addition of 1 or 10 mm of MgCl_2_, ZnCl_2_, CoCl_2_, NiCl_2_, or MnCl_2_. WT activity was standardized to 100%, and background signals were subtracted from WT reactions without UDP-GlcNAc. Reactions were performed in triplicate. The apparent *K_m_* and *k*_cat_ for UDP-GlcNAc and 4MU-GlcNAc were determined by Michaelis-Menten kinetics using varying concentrations of one substrate in the presence of an excess of the other substrate. Data were analyzed in the GraphPad Prism program. *Sm*NodC point mutants were assayed using the protocol developed for WT *Sm*NodC in the presence of 10 mm MgCl_2_. Nikkomycin Z was purchased from Sigma.

## RESULTS AND DISCUSSION

### 

#### 

##### SmNodC Shows Kinetic Properties Similar to ScCHS2

**FIGURE 2.**
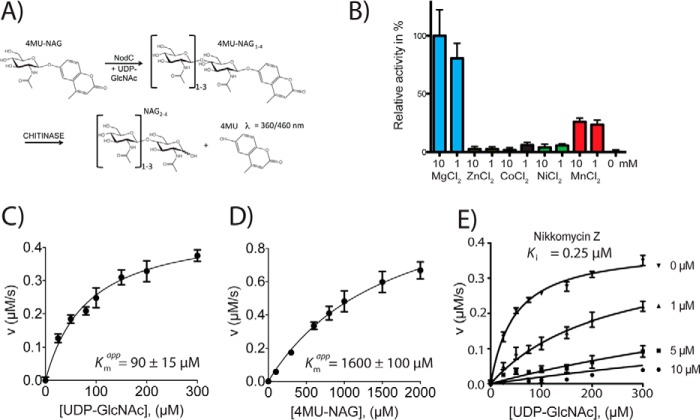
***Sm*NodC activity assay, metal dependence, Michaelis-Menten kinetics, and inhibition.** *A*, novel *Sm*NodC activity assay. NodC is able to elongate the acceptor sugar 4MU-GlcNAc (4MU-NAG) on its non-reducing end 4-OH to yield 4MU-GlcNAc_2–4_. This product (but not 4MU-GlcNAc) is a chitinase substrate (33) releasing fluorescent 4MU. *B*, *Sm*NodC metal dependence was investigated in the presence of 1 and 10 mm Mg^2+^, Zn^2+^, Co^2+^, Ni^2+^, and Mn^2+^. The highest relative activity was achieved in the presence of 10 mm MgCl_2_ (standardized to 100% relative activity) and 1 mm of MgCl_2_ (80% relative activity). Mn^2+^ was able to activate *Sm*NodC, whereas no activation was observed with Zn^2+^, Co^2+^, and Ni^2+^. *C* and *D*, Michaelis-Menten kinetics of *Sm*NodC. *K*_*_m_*_^app^ of *Sm*NodC WT were determined for both substrates, UDP-GlcNAc (*C*) and 4MU-GlcNAc (*D*), in triplicate, in the presence of 10 mm MgCl_2_. The velocities (micromolar per second) are shown as a function of the concentration of the second substrate. The second substrate was used in excess in each kinetic measurement, and less than 20% was utilized during the reaction (linear range). *E*, *K_i_* determination of Nikkomycin Z against *Sm*NodC. Data were fitted using the non-linear fit of transformants in the GraphPad Prism program. The substrate concentration of UDP-GlcNAc was varied to determine a competitive inhibition mode of Nikkomycin Z to UDP-GlcNAc. 4MU-GlcNAc was used at a concentration of 1.5 mm, corresponding to the *K_m_* value. The experiment was performed in triplicate.

Full-length *Sm*NodC was cloned and overexpressed in *E. coli* as a C-terminal mCherry fusion protein. Membrane fractions were prepared, and the protein was found to be expressed as a stable mCherry fusion into the bacterial membrane. *Sm*NodC activity was initially determined using a traditional thin layer chromatography assay (24). We found that *Sm*NodC was able to utilize the fluorogenic compound 4MU-GlcNAc as the acceptor substrate, forming chitooligosaccharides with 4MU capping the reducing end. This allowed us to develop a novel, non-radioactive, high throughput screen-compatible, coupled assay that makes use of the specific hydrolysis of 4MU-(GlcNAc)_n_ oligomers on the reducing end by a chitinase to release fluorescent 4MU (Fig. 2*A*). This novel assay is a fast and sensitive two-step assay for the determination of shorter chitooligosaccharide products. Like CHS, NodC are thought to be metal dependent enzymes (21). Indeed, *Sm*NodC is maximally active in the presence of 10 mm MgCl_2_, whereas treatment with EDTA prevents *Sm*NodC activity, presumably by chelating the divalent cation, which is bound in the active site (Fig. 2*B*). The apparent *K_m_* for UDP-GlcNAc is 90 ± 15 μm, and the apparent *K_m_* for 4MU-GlcNAc is 1.6 ± 0.4 mm (Fig. 2, *C* and *D*). This is similar to the apparent *K_m_* of UDP-GlcNAc for yeast chitin synthases (8, 25, 26).

##### SmNodC Is Inhibited by a Chitin Synthase Inhibitor

To further investigate the suitability of *Sm*NodC as a model for fungal chitin synthases, we studied the susceptibility to the chitin synthase inhibitor Nikkomycin Z (Fig. 2*E*). Nikkomycin Z inhibits yeast chitin synthases in the 0.2–1000 μm range (27). Interestingly, Nikkomycin Z is a competitive inhibitor with the substrate UDP-GlcNAc, with a *K_i_* of 0.25 μm. This further highlights the suitability of *Sm*NodC as a model for fungal chitin synthases, implying conservation of the active site in agreement with sequence alignments (Fig. 1). It further suggests that, together with the novel *Sm*NodC assay, this system may offer new opportunities for the high throughput screen-based discovery of new CHS inhibitors.

##### The Topology of SmNodC Matches the CHS Core

**FIGURE 3.**
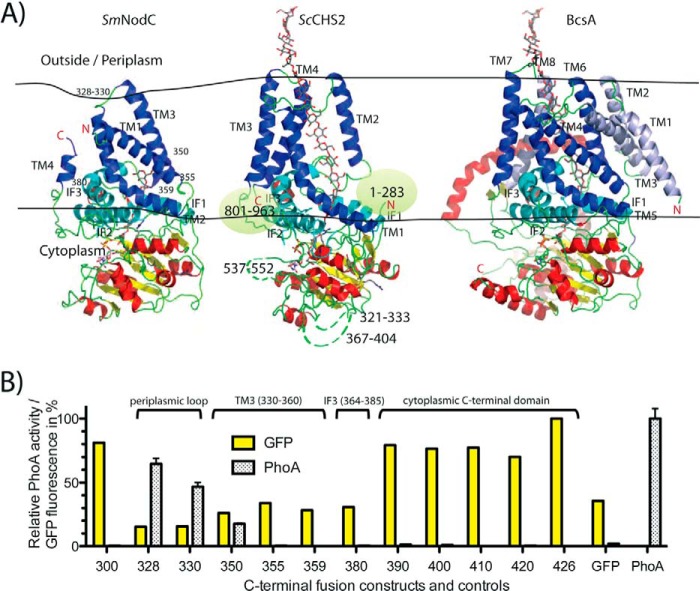
**Structural model of *Sm*NodC and *Sc*CHS2 and topology mapping of *Sm*NodC.** *A*, structural model of *Sm*NodC and *Sc*CHS2. Shown is a schematic of BcsA (PDB code 4HG6, Ref. 5) and corresponding structural models calculated for *Sm*NodC and *Sc*CHS2. The membrane is indicated by two *black lines*. Structurally conserved TM and IF domains are labeled and colored as in Fig. 1. TM domains are colored in *blue*, and cytoplasmic interface leaning domains are colored in *cyan*. Non-conserved BcsA TM1–3 are shown in *light blue*. The structurally conserved N-terminal catalytic GT-A fold domain is shown with *red* α-helices, *yellow* β-strands, and *green* loops. *B,* topology mapping of *Sm*NodC using GFP and PhoA fusions. Twelve C-terminal fusion constructs were cloned, expressed, and investigated for their GFP fluorescence and PhoA activity. The numbers on the *x* axis correspond to the C terminus of the construct. Signals were standardized to 100% for the highest signal, and fusion constructs with GFP fluorescence greater than 20% and a PhoA activity greater than 20% were considered positive results. *Sm*NodC contains only a small loop between TM2 and TM3 that is located in the periplasm. The catalytic core (30–300) and the C-terminal domain (355–426) are clearly located in the cytoplasm. GFP fusion control protein FrdC contains a cytoplasmic C terminus, and PhoA control protein TarA has its C terminus located in the periplasm (23).

Morgan *et al.* (5) have recently reported the first structure of a processive glycosyltransferase from the GT-2 family, BcsA, a bacterial cellulose synthase from *Rhodobacter sphaeroides* (Fig. 3*A*), suggesting a catalytic mechanism of cellulose synthesis and transport across the bacterial membrane. They further reported insights into the positioning and potential function of the CON1/CON2 motifs conserved across GT-2 family members and suggested key residues that are located in the active site and might be important for catalytic activity (5). We combined the BcsA crystal structure and sequence alignments (Fig. 1) to generate structural models of *Sm*NodC and *Sc*CHS2 (Fig. 3*A*) to serve as a guide for the experimental verification of *Sm*NodC topology and to probe the function of conserved residues by mutagenesis. Structural models of *Sm*NodC and *Sc*CHS2 were generated with the RaptorX server (28), using the BcsA structure as a template. The *Sm*NodC model was built with a 62% overall alignment score for the full-length protein and a 76% alignment score for the catalytic core (residues 46–284). The *Sm*NodC structural model suggests three TM domains (Cys-4 to Lys-28, Leu-303 to Ile-329, and Trp-336 to Leu-360) and three cytoplasmic interface-leaning domains IF1 (187–202), IF2 (271–291), and IF3 (364–385) (Figs. 1 and 3*A*).

To probe this predicted topology, we employed GFP and PhoA C-terminal fusion proteins to distinguish between the cytoplasmic and periplasmic regions of *Sm*NodC. PhoA is only active when located in the periplasm, and GFP/mCherry is known to only properly fold and, thus, fluoresce in the bacterial cytoplasm (23, 29). All constructs that do have their C terminus in the cytoplasm will have a fluorescent GFP fusion (23) and will not show PhoA activity.

Full-length (1–426) *Sm*NodC expresses as a fluorescent mCherry fusion protein in *E. coli*, whereas a PhoA fusion does not possess any activity (Fig. 3*B*), suggesting that the C terminus is facing the cytoplasm, in agreement with the structural model (Fig. 3*A*). This is in contrast to an earlier study of NodC topology (22) where PhoA-fusion on a C-terminally truncated construct showed PhoA activity, which would indicate that the C terminus of NodC is located in the periplasm. We constructed a further 11 C-terminal *Sm*NodC truncations, each with a C-terminal PhoA or GFP fusion. Fig. 3*B* shows all *Sm*NodC-PhoA/GFP fusion proteins constructed and their fluorescence/PhoA activity. On the basis of GFP fluorescence (present) and PhoA activity (absent), residue Ser-300, which is part of the active site, is located in the cytoplasm, in agreement with the structural model (Fig. 3, *A* and *B*). The structural model predicts that the first residues after the cytoplasmic catalytic core (46–284) in the periplasm are Leu-328 to Met-330 (Fig. 3*A*). In agreement with this, the C-terminal PhoA fusion of the 1–330 truncation is active (Fig. 3*B*). Furthermore, no C-terminal truncation after residue 330 shows detectable PhoA activity, whereas NodC-GFP fusions at Cys-350, Leu-355, Gln-359, and Lys-380 are fluorescent (Fig. 3*B*). This is in agreement with the structural *Sm*NodC model that predicts residue range 360–380 to form a cytoplasmic interface leaning domain (IF) and not a TM domain. This domain sits on top of IF2 (271–290) and closely interacts with TM2 (Fig. 3*A*). Our topology studies did not cover IF1 (187–202) and IF2 (271–291), which did not show significant sequence homology with BcsA and are, therefore, not predicted accurately by the structural model, although IF1-IF3 show good sequence similarity among *Sm*NodC and *Sc*CHS2 (Fig. 1).

Taken together, the experimental topology mapping approach (Fig. 3*B*) validates the *Sm*NodC topology as derived from the structural model (Fig. 3*A*), showing that NodC enzymes contain three transmembrane domains that traverse the membrane in an out-in, in-out, out-in fashion, connected by a cytoplasmic hydrophilic catalytic core located between TM1 and TM2 and by a short periplasmic hydrophilic loop between TM2 and TM3 (Fig. 3*A*). The fourth domain is predicted to be a cytoplasmic interface leaning domain. This topological arrangement positions the catalytic domain into the cytoplasm, allowing the enzyme to access the cytoplasmic pool of the sugar nucleotide substrate UDP-GlcNAc.

##### The Catalytic Machinery of SmNodC/ScCHS2 Consists of Three Conserved Motifs

**FIGURE 4.**
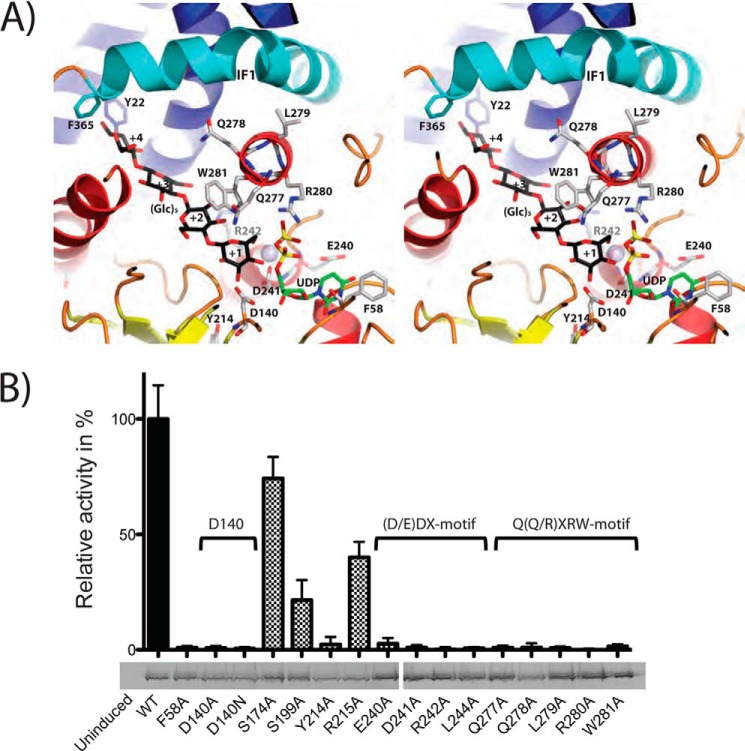
***Sm*NodC active site view and point mutations probing the catalytic mechanism.** *A*, the *Sm*NodC active site in complex with pentacellulose. Shown is a stereo figure of the *Sm*NodC active site. Residues that were investigated by mutagenesis in this study are shown as *sticks* with *gray* carbon atoms, *red* oxygen atoms, and *blue* nitrogen atoms. The active site contains the product UDP (*green* carbon) and the superimposed Glc_5_ from the ternary BcsA complex with *black* carbon atoms (5). The Mg^2+^ atom was placed on the basis of a superposition of the SpsA Mg^2+^-Mn^2+^ complex and is shown as a *blue sphere* (PDB code 1QGQ) (30). *B*, *Sm*NodC and *Sc*CHS2 point mutants inactivate GT-2 activity. The CHS-motifs D, D*X*D, Q(Q/R)*X*RW, and additional conserved and non-conserved residues were mutated in *Sm*NodC to probe the involvement of these residues in polysaccharide synthesis. Relative *Sm*NodC activity of the mutants was found to correlate with previously described *Sc*CHS2 mutants (8) In-gel-fluorescence reveals that all mutants were expressed and inserted into the membrane at similar levels.

The BscA structure was crystallized in complex with UDP and a cellulose product, facilitating the interpretation of the predicted active site in the *Sm*NodC model. To probe the role of active site residues, we designed specific point mutants and tested the effects on chitooligosaccharide synthesis. According to the sequence alignment (Fig. 1), *Sm*NodC Phe-58 corresponds to Tyr-148 in cellulose synthase and appears to form a hydrophobic stacking interaction with the UDP-GlcNAc uracil moiety (Fig. 4*A*). In agreement with this, the F58A mutation inactivates *Sm*NodC (Fig. 4*B*), similar to the equivalent mutation (Y298A) in *Sc*CHS2 (8). The structural model further shows that the side chain of *Sm*NodC Asp-140 (Asp-441 in *Sc*CHS2) is in proximity to the side chain of *Sm*NodC Asp-241 (Asp-562 in *Sc*CHS2), coordinating the Mg^2+^ ion that is essential for catalysis (Fig. 4*A*). Mutation of either of these residues to alanine or asparagine inactivate the enzyme (Fig. 4*B*), similar to equivalent mutations of these residues in *Sc*CHS2 (8).

The conserved Q(Q/R)*X*RW CON1 motif, characteristic of processive GT-2 enzymes, forms an α-helix lining the active site (Fig. 4*A*). Mutations of any of these residues to an alanine inactivate *Sm*NodC (Fig. 4*B*), similar to the corresponding *Sc*CHS2 mutations (8). The model suggests that the invariant residues **Q**(Q/R)*X***RW** are predicted to face the active site, whereas the invariant Q**(Q/R)*X***RW residues are positioned on the back of this helix (Fig. 4*A*). Trp-281 of *Sm*NodC (Trp-605 in *Sc*CHS2) is potentially involved in π-π stacking interactions with the GlcNAc sugar in the +2 subsite (Fig. 4*A*). This is in agreement with kinetic data showing that mutation of W281A (Trp-605 in *Sc*CHS2) abolishes catalytic activity (Fig. 4*B*). Similarly, the side chain of Arg-280 (Arg-604 in *Sc*CHS2) is predicted to bind the negatively charged diphosphate moiety of UDP-GlcNAc (Fig. 4*A*), consistent with the observation that the alanine mutants of this residue in *Sm*NodC (Fig. 4*B*) and *Sc*CHS2 are inactive (8).

According to the reaction mechanism proposed for processive, inverting glycosyltransferases, two side chain carboxylates are required (30). These have been proposed to act as the general base, abstracting the proton of the acceptor substrate, and the second carboxylate assists to coordinate the leaving group (Mg^2+^) departure (30) (*Sm*NodC Asp-140). It is not known precisely how the catalytic base is regenerated (deprotonated), but this presumably, ultimately, involves the transfer of a proton to a water molecule.

NodC and class II CHS share a conserved EDR motif (Fig. 1, *SmNodC 240–242* and *ScCHS2 561–563*). The *Sm*NodC model reveals that the side chain of Asp-241 is positioned ∼ 4.0 Å from the hydroxyl group of the acceptor sugar (Fig. 4*A*). This aspartate may therefore act as the catalytic base, activating the sugar acceptor for nucleophilic attack. Glu-240 and Tyr-214 are in proximity to Asp-241, perhaps tuning its p*K_a_* (Fig. 4*A*). The mutation of any of these conserved residues inactivates *Sm*NodC and *Sc*CHS2 (Fig. 4*B*) (8). Mutation of Arg-215, which is seen to position Tyr-214 in the *Sm*NodC model, reduces the relative catalytic activity to ∼40%. With the help of the structural model, we propose that the side chain of this conserved arginine is important, but not essential, for the structural integrity of the GT-A folded catalytic domain. Mutation of the final residue of the EDR motif, Arg-242 in *Sm*NodC (Arg-563 in *Sc*CHS2), and mutation of Leu-244 in *Sm*NodC (Leu-565 in *Sc*CHS2) inactivate the enzyme (Fig. 4*B*) (8). The *Sm*NodC model suggests that these residues may be required for precise positioning of the α-helix with the catalytic EDR motif (Fig. 4*A*). Two control mutations away from the active site, S506A and S174A (surface-exposed, minor reductions in activity, Fig. 4*B*) did not affect the catalytic activity.

##### The SmNodC Active Site Forms a Molecular Ruler

**FIGURE 5.**
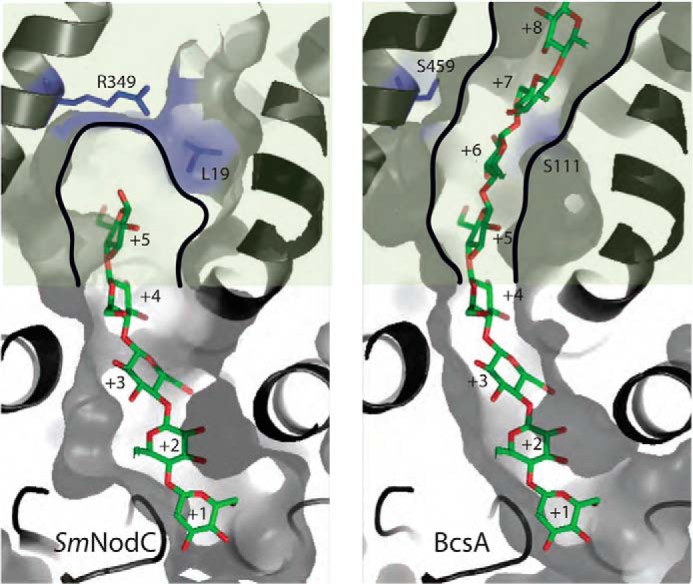
**Product-binding sites of *Sm*NodC and BcsA.** Surface and secondary structure representation of the product-binding sites for *Sm*NodC (*left panel*) and BcsA (*right panel*) (5). Protein domains that are predicted to be membrane-embedded are colored in *light green*. The polysaccharide represents the chitooligosaccharide in *Sm*NodC and is shown as a *stick* representation with *green* carbon atoms and *red* oxygen atoms. The predicted product-binding site for *Sm*NodC is limited by Arg-349 and Leu-19 to five binding sites (+1 to +5), whereas the cellulose synthase structure forms a transport channel (*black lines*) through the membrane formed by the transmembrane domains (Ser-111 and Ser-459).

One of the key differences between *Sm*NodC and *Sc*CHS2/BscA is that *Sm*NodC synthesizes only short, soluble chitooligosaccharides, up to (GlcNAc)_5_, whereas *Sc*CHS2/BscA produces long, insoluble polysaccharides that are deposited in the cell wall. Interestingly, a previous study with chimeras of NodC enzymes from different species identified that the C terminus beyond Ile-262 regulates chitooligosaccharide product length (31). The *Sm*NodC model reveals that the C-terminal domain (Ile-262 until the C terminus) indeed forms the product-binding site (Figs. 3*A* and 4*A*). The product-binding site is limited by a key residue, Arg-349, which is predicted to point toward Leu-19 and defines a molecular ruler for the synthesis of chitopentaose (Fig. 5). In BcsA, longer products can be synthesized because these residues are both serines that form a proper transport channel across the membrane (Fig. 5). Furthermore, *Sm*NodC lacks additional transmembrane domains to form a transport channel across the lipid bilayer (Fig. 3*A*).

##### Mechanism of Chitin Synthesis and Transport

**FIGURE 6.**
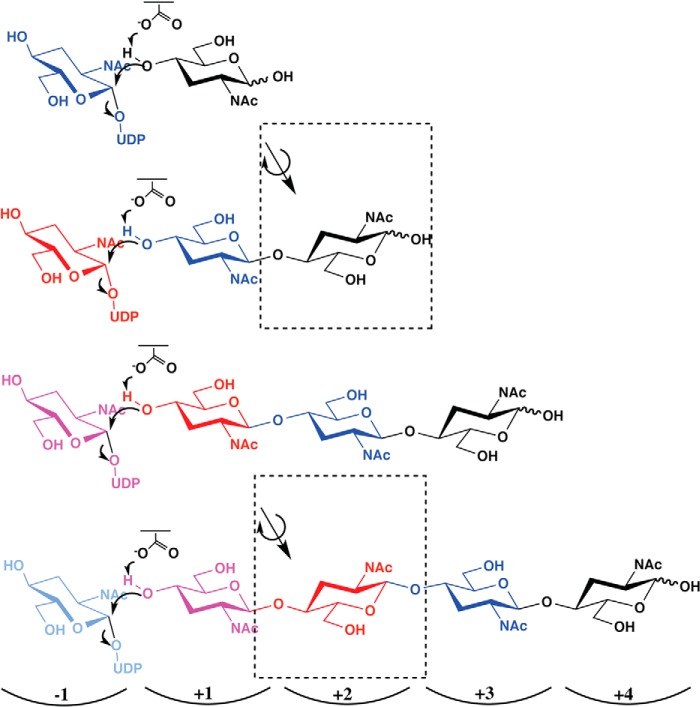
**Proposed reaction mechanism of chitin synthesis.** Chemical drawing of the proposed reaction mechanism for NodC and chitin synthesis. The 1-hydroxyl group of the donor substrate UDP-GlcNAc is transferred onto the non-reducing end of the growing acceptor oligosaccharide. Asp-241 (*Sm*NodC, Asp-562 in *Sc*CHS2) may act as the catalytic base, activating the sugar acceptor for nucleophilic attack. When the transfer reaction is completed, UDP leaves the active site. In the first synthesis step, the two terminal sugars (-1 and +1) of the growing chain would both rotate while moving into the next binding site (+1 and +2). During further elongation, the +1 sugar would only rotate every second synthesis step (*red* sugar compared with *blue* sugar). All sugars moving into the +3/+4 subsites would remain of a fixed orientation. This rotation and translocation enables the newly added non-reducing sugar to be in the same acceptor position as the previous one.

We identified catalytic residues by structural modeling and mutagenesis. It is possible to predict the *Sm*NodC product-binding subsites (+1 to +5) of the acceptor polysaccharide by superimposing the glucose polymer from the BcsA structure onto the *Sm*NodC model (Figs. 4*A* and Fig. 5). NodC enzymes synthesize polysaccharides of up to chitin pentaose, whereas *Sc*CHS2 and BcsA synthesize long polysaccharides. The main product of *Sm*NodC is chitotetraose (31). The product-binding tunnel reveals distinct binding sites that accommodate the *N*-acetyl moiety of the chitooligosaccharide (Fig. 5). Together with the data on the catalytic residues, this allows us to propose a reaction mechanism for processive chitooligosaccharide synthesis. The acceptor GlcNAc moiety sits in the +1 site with the 4-hydroxyl group pointing toward the UDP-GlcNAc binding site (Fig. 6). The acceptor sugar is activated by the catalytic base (Asp-241 in *Sm*NodC) and performs a nucleophilic attack on the anomeric carbon of UDP-GlcNAc, generating a β-(1,4)-glycoside. Upon completion of the transfer reaction, UDP leaves the active site. At the same time the +1 sugar moves into the +2 pocket, whereas the newly added GlcNAc moves and rotates into the +1 site. This produces an acceptor sugar position/confirmation that is identical to the first step. An alternative is the mechanism proposed for BscA, which does not involve sugar rotation, resulting in the next 4-hydroxyl acceptor approaching the catalytic center from the opposite site than the acceptor in the previous step (5). In the mechanism proposed here, sugars translocating from the +1 into +2 subsite would rotate every second synthesis step, whereas sugars translocating into the +3/+4 subsites could remain of a fixed orientation, requiring these subsites to accommodate the *N*-acetyl moieties in both sugar conformations. Energy for translocation/rotation may be supplied as steric strain on the +1 sugar introduced with each processive glycosyltransferase step.

## CONCLUSIONS

*Sm*NodC was overexpressed in *E. coli* cells as an active mCherry-His tag fusion protein. We developed a novel, efficient, high throughput-compatible, non-radioactive assay that allowed us to determine Michaelis-Menten kinetics and metal dependence. With the help of this assay, we were able to show that the CHS inhibitor Nikkomycin Z is a potent competitive NodC inhibitor. Therefore, we propose that *Sm*NodC is a useful model to screen for small molecule inhibitors and to identify novel molecules that might also inhibit the fungal chitin synthases.

Structural insights into the active site of *Sm*NodC and comparing conserved active site residues between *Sm*NodC and *Sc*CHS2 reveals that both enzymes share a very similar nucleotide sugar- and acceptor-binding site. The topological arrangement was validated by biochemical experiments and revealed that NodC enzymes contain three membrane-spanning and three cytoplasmic interface-leaning domains, similar to the recently determined structure of a cellulose synthase (5), and the predicted CHS structure. Furthermore, our topology studies correlate with eukaryotic GT-2 enzymes (5, 32). *Sc*CHS2 contains six TM domains, four of which can be structurally modeled on the basis of the bacterial cellulose synthase structure (Fig. 3*A*). The remaining two TM domains are predicted to be part of a chitin transport channel that is absent from *Sm*NodC because this enzyme only synthesizes short chitooligosaccharides for further processing in the cytoplasm.

Amino acids conserved between *Sm*NodC and CHS were targeted by mutagenesis. The *Sm*NodC mutants show inactivation, similar to the corresponding *Sc*CHS2 mutants. On the basis of these data, we propose a reaction mechanism where the only sugar that rotates is the +1 sugar because it translocates to the +2 subsite, and only every second step. This preserves the up/down arrangement of the *N*-acetyl groups as seen in crystalline chitin and consistently presents the 4-OH hydroxyl of the growing acceptor to the active site in the same orientation.

NodC enzymes lack the transmembrane domains that are present in BcsA and CHS2 to form a product translocation channel across the lipid bilayer. Our structural model further revealed that the product-binding site of *Sm*NodC is defined by two residues that are conserved among NodC enzymes. These define a molecular ruler to synthesize chitooligosaccharides of limited length, whereas CHS encode a chitin transporter channel presumably build from at least four TM domains.

We propose *Sm*NodC as a model system to study chitin synthases on a molecular and structural level to elucidate the reaction mechanism on a structural level of chitooligosaccharide synthases and to identify novel CHS inhibitors because we propose that novel inhibitors of NodC enzymes will be interesting lead compounds to inhibit chitin synthases.

## Footnotes

3 The abbreviations used are:

CHS: chitin synthase(s)
TM: transmembrane
*Sc*CHS2: *S. cerevisiae* CHS2
SmNodC: *S. meliloti* NodC
PhoA: alkaline phosphatase
4MU: 4-methylumbelliferyl
IF: interface leaning domain
BcsA: *R. sphaeroides* cellulose synthase
CON: conserved region
GT: glycosyl transferase.
